# Facilitators and barriers to collaboration between pre-hospital emergency and emergency department in traffic accidents: a qualitative study

**DOI:** 10.1186/s12873-023-00828-4

**Published:** 2023-05-29

**Authors:** Hasan Jamshidi, Reza Khani Jazani, Ali Khani Jeihooni, Ahmad Alibabaei, Shahram Alamdari, Majid Najafi Kalyani

**Affiliations:** 1 Department of Nursing, School of Nursing, Fasa University of Medical Sciences, Fasa, Iran; 2 Department of Health in Disaster and Emergencies, School of Health, Safety and Environment, Shahid Beheshti University of Medical Sciences, Tehran, Iran; 3 Nutrition Research Center, Department of Public Health, School of Health, Shiraz University of Medical Sciences, Shiraz, Iran; 4 School of Health, Safety and Environment, Shahid Beheshti University of Medical Sciences, Tehran, Iran; 5 Research Institute for Endocrine Sciences, Obesity Research Center, Shahid Beheshti University of Medical Sciences, Tehran, Iran; 6 Department of Nursing, School of Nursing and Midwifery, Shiraz University of Medical Sciences, Shiraz, Iran

**Keywords:** Pre-hospital emergency, Emergency department, Collaboration, Traffic Incident, Qualitative research

## Abstract

**Background:**

Death caused by traffic accidents is one of the major problems of health systems in low- and middle-income countries. Rapid handover of the traffic accident victims and proper collaboration between the pre-hospital and emergency departments (EDs) play a critical role in improving the treatment process and decreasing the number of accidental deaths. Considering the importance of the collaboration between pre-hospital and emergency departments, this study was designed to investigate the facilitators and barriers of collaboration between pre-hospital and emergency departments in traffic accidents.

**Method:**

This research is a qualitative study using content analysis. In order to collect data, semi-structured interviews were used. Seventeen subjects (including pre-hospital and emergency department personnel, emergency medicine specialists, and hospital managers) were selected through purposive sampling and were interviewed. After transcribing and reviewing interviews, data analysis was performed with the qualitative content analysis approach.

**Results:**

The participants consisted of 17 individuals (15 persons in pre-hospital and emergency departments with at least three years of work experience, one emergency medicine specialist and one hospital manager) who were selected by purposive sampling. The interviews were analyzed and three main categories and seven sub-categories were extracted. The main categories included “individual capabilities”, “development of mutual understanding”, and “infrastructures and processes”.

**Discussion:**

Proper and practical planning and policymaking to strengthen facilitators and eliminate barriers to collaborate between pre-hospital and emergency departments are key points in promoting collaboration between these two important sectors of health system and reducing the traffic accident casualties in Iran.

## Background

**Fig. 1:**
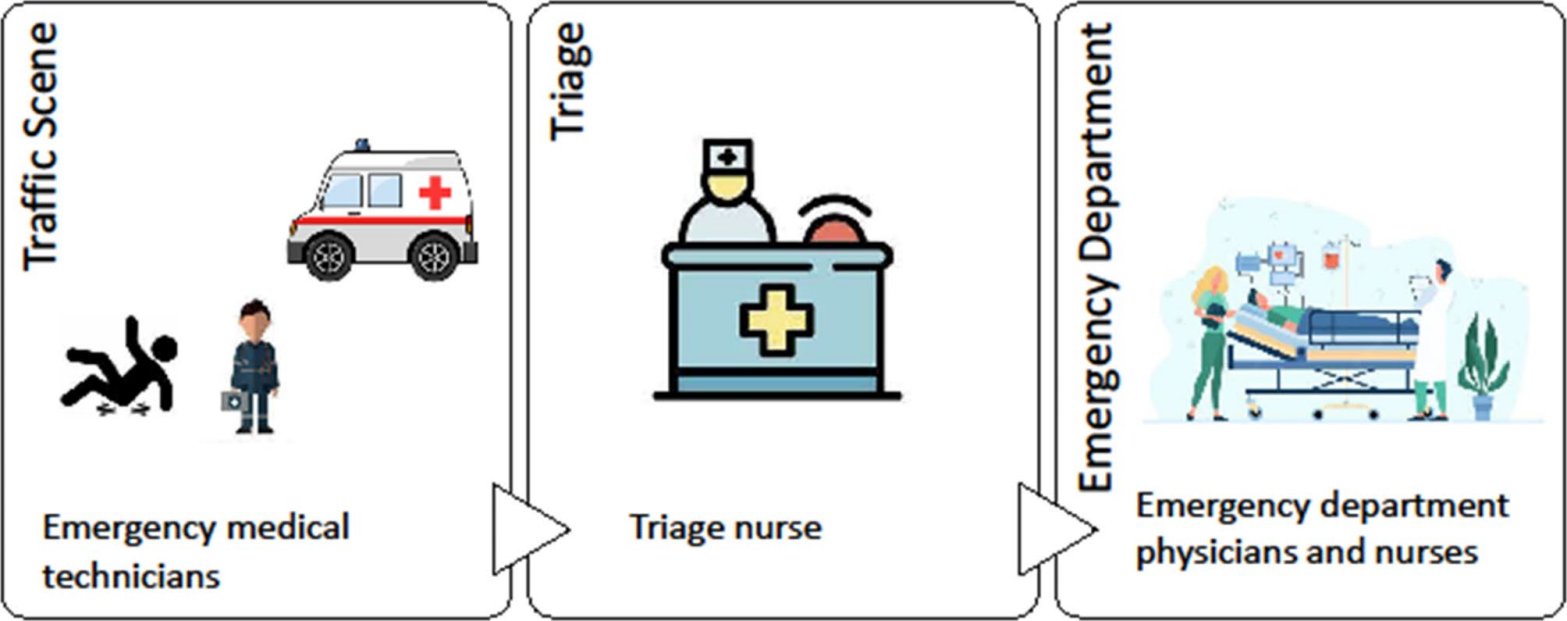
The process of traffic victims’ handover in Iran

Traffic accidents kill about 1.2 million people and injure or disable 20 to 50 million individuals annually, accounting for 25% of the world’s deaths and 22% of the world’s disabilities. They are also the major challenge facing the global health system [1], as well as the health systems in low and middle income countries. Iran with 28,000 deaths per year has the first rank in the world in terms of the frequency of fatalities related to driving [2–4]. Road accidents in Iran are 20 times the world average and the second leading cause of death [3]. In Iran, the pre-hospital emergency department is responsible for providing primary health care and delivering the injured from traffic accidents to the emergency department, where usually one-third of its beds are occupied by road accident victims [5, 6]. The process of traffic victims’ handover is illustrated in Fig. 1.

1. Initially, emergency medical technicians (EMTs) would be present at the accident scene and perform basic resuscitation measures on the victims of traffic accidents.
2. Then, the injured are handed over to the nurse in charge of triage in the hospital’s emergency department.
3. After being admitted to the hospital, the injured are visited by an emergency medicine specialist and the necessary measures are taken.

In the event of an accident, intra-departmental and inter-departmental collaboration is of great importance, and the providing effective services requires the collaboration of all health system staff [7, 8]. According to a number of studies, one of the factors causing high mortalities in traffic accidents is the lack of proper collaboration between pre-hospital departments and emergency departments in many developing countries [9]. Accelerating patient handover, facilitating the continuation of treatment process, reducing mortality, and increasing satisfaction are the results of effective collaboration between pre-hospital departments and emergency departments [9–13].

Despite the great importance of collaboration and its significant impacts on reducing mortalities of road accidents [14], few quantitative studies have been conducted in this field [15, 16]. A majority of studies conducted in Iran have also focused on the quantity and causes of delay in starting pre-hospital care or management factors [17]. Erie et al. reported the lack of proper collaboration between emergency department staff and other organizations as one of the challenges experienced by pre-hospital emergency staff and believed that they need to collaborate with physicians, nurses, midwives, and psychotherapists to promote care in patient handover [18]. The promotion of pre-hospital and emergency department collaboration requires identifying their challenges and problems. In this regard, identifying the experiences of the personnel involved in this process seems to be a great contribution. A comprehensive understanding of all aspects of this phenomenon is needed to identify facilitators or barriers affecting the collaboration between pre-hospital and emergency departments. Since neither quantitative studies nor one or more questionnaires are sufficient to approach such an in-depth understanding, the present qualitative study was conducted to identify facilitators and barriers of collaboration between the pre-hospital and emergency departments.

## Materials and methods

### Methodology and Population of the study

The present study is a qualitative research based on the content analysis approach. This study was conducted on 17 healthcare professionals affiliated with Fasa University of Medical Sciences including seven emergency medical technicians (EMTs), six nurses (triage and emergency department nurses), two general physicians, one emergency medicine specialist, and one hospital manager. The data were collected from two pre-hospital emergency centers in Fasa City, and Valiasr hospital emergency department in Fasa, Fars, Iran. This major hospital is a trauma center receiving more than 2000 traffic accident victims annually.

The criteria for entering the study were having at least 3 years of work experience, having rich experience, and willingness to participate in the study. Purposive sampling initiated in 2015 and continued with theoretical sampling and individual in-depth individual interviews until data saturation was reached.

### Data collection and analysis

Semi-structured face-to-face interviews were used to collect the data. All interviews began with an open question, such as “Talk about a day at work and your collaboration with the emergency department staff.“ and some guiding questions like “Please give me an example.“ were also used to further clarify the topic. During the interviews, follow-up questions were asked to clarify the concepts. On average, the interviews lasted for 50 min. After explaining the purpose and the method of the interview and obtaining the participants’ informed consent regarding the recording of their speeches, the interviews were recorded and then transcribed verbatim by the researchers. The interviews were conducted by three qualitative research experts. The data were then reviewed several times to reach an overall understanding.

Two of the three researchers reviewed the data independently using standard content analysis methods, extracting semantic units from the interview statements (including words, sentences, and paragraphs), and coded them based on their similarities and differences. According to continuous thinking, interpretation, and comparison of data, key categories and themes were extracted and primary categories were identified. The final categories were extracted by summarizing the concepts and codes and according to the differences or similarities of the initial categories [19].

The interview was developed for this study has previously been published [20].

The validity of this study was obtained by using continuous comparison methods and observation by the research team and external observers. Credibility was obtained through researcher’s long-term engagement, the combination of data collection, repeated reviews, supervisor’s reviews, and continuous comparison of data. External member checks were used to achieve dependencies. Manuscripts and notes were handed to two associated professors, who approved the confirmability of the findings. Finally, the transferability of the present study was approved due to the description of the rich data [19, 21]. Some ethical considerations included confidentiality of information, written informed consent form for interviews and interview records, and the right to withdraw from the study whenever the participants wanted, were considered. The study was approved by the Ethics Committee of Shahid Beheshti University of Medical Sciences.

## Results

The participants in this study were 17 individuals (13 people in pre-hospital and emergency departments with at least three years of work experience, two general physicians, one emergency medicine specialist, and one hospital manager) who were selected through purposive sampling. The average age of the participants was 35 years and their average work experience was eight years. According to the data analysis, three main categories (individual capabilities, development of mutual understanding, and infrastructures and processes) were extracted. Table 1 summarizes the three main categories and seven sub-categories we derived, along with a representative quotation for each of them (Table 1).

**Table 1:** The three main categories and seven sub-categories extracted, along with representative quotations

Individual Capabilities	Individual Knowledge	“When trained emergency staff handover a patient, they well-express the patient’s history and problems so that the recipient feels comfortable.”
	Individual Experience	“I am more comfortable with someone with better and longer work experience because he or she knows better what is important when handing over a traffic accident injured.“
Development of Mutual Understanding	Common Educational Programs	“The personnel who participate in joint workshops have a better collaboration because of a greater understanding of each other’s problems.”
	Sharing Experiences	“Staff who have experience in both pre-hospital and emergency departments better understand the current state of a person who has just been injured in an accident and is to be handed over.“
	Empathic Behaviors	“Our friendship allows us to deliver and admit patients in a shorter time since we do not pay attention to trivial matters.”
Infrastructures and Processes	Adequate and Similar Equipment	“I do not have the opportunity to directly connect to the hospital from the accident scene. I cannot report the number of injured and the type of injuries to the hospital from the accident scene for them to be prepared.”
	Defects and Disorganizations	“The form containing the written emergency report and patient’s history does not contain a number of important issues. For example, no blood sugar level is included for a patient with a low level of consciousness.”

### 1. Individual capabilities

“Individual capabilities” was the first extracted category and consisted of two subcategories: “individual knowledge” and “individual experience.” The individual capabilities provide opportunities for staff to be more scientifically and empirically efficient and to demonstrate better interactive responsiveness in inter-sectorial collaboration and patient delivery to facilitate the collaboration process.

A. Individual knowledge.

The results indicated that the more successful the individuals were in acquiring knowledge individually, the more effectively they played their role in inter-sectorial collaboration. Higher levels of knowledge and being scientifically updated provide the basis for more effective and facilitated collaboration. Having undergraduate or higher educational records had provided the knowledge for these staff. In this regard, one of the physicians from emergency department stated, “When trained emergency staff handover a patient, they well-express the patient’s history and problems so that the recipient feels comfortable. In fact, personnel’s high levels of education make the patient handover less difficult.“

Another emergency medical technician from the pre-hospital emergency department said, “The trained personnel in the triage department ask many questions regarding the condition of the injured, the mechanism of the injury, and the patient’s affected organs. They also check symptoms. In this way, I feel comfortable when delivering the injured to them.“

B. Individual experience.

The results indicate that the more experienced the staff are, the more effective the inter-sectorial collaboration is, and that the higher level of experience as a facilitator provides the basis for more effective collaboration.

For instance, one emergency department nurse, with eight years of work experience mentioned, “I am more comfortable with someone with better and longer work experience because he or she knows better what is important when handing over a traffic accident injured.“

The most important foundations for the emergence of inter-sectorial collaboration were studying scientific books related to the field, having theoretical knowledge, and practical and field experiences.

### 2. Development of mutual understanding

The development of the mutual understanding was the second category containing three subcategories: “common educational program”, “sharing experiences”, and “empathic behaviors”. Developing mutual understanding provides opportunities for scientific, skillful, attitudinal and behavioral closeness of personnel and facilitates the collaboration process and vice versa.

A. Common educational programs.

The participants considered their joint programs important and emphasized on its effective role in facilitating collaboration. One emergency medical technician quoted, “The emergency department holds a monthly educational class on topics like trauma or transportation. We have more understanding and collaboration with the hospital staff who participate in these classes”. Another participant also noted, “The personnel who participate in joint workshops have a better collaboration because of a greater understanding of each other’s problems.”

From the participants’ point of view, strengthening the areas of developing joint educational programs, including joint classes and workshops, plays a key role in facilitating collaboration between the pre-hospital and emergency departments.

B. Sharing experiences.

Sharing experiences also facilitates collaboration. One of the emergency department staff said, “Staff who only have a working experience in a hospital triage cannot understand the current state of a person who has just been injured in an accident and is to be handed over; however, those who have experience in both pre-hospital and emergency departments better understand this condition.“

From the participants’ perspective, sharing experiences through having joint operational maneuvers and familiarizing personnel with the difficulties and complexities of work in both departments are crucial in facilitating collaboration between the pre-hospital and emergency departments.

C. Empathic behaviors.

Empathic behaviors, mutual understanding, and creating a friendly atmosphere in the work environment were other facilitators of collaboration, which were emphasized by the participants. The results indicated that respectful, professional, and friendly behaviors make inter-sectorial collaboration more effective. In these places, which are filled with engagement and friendship, solving possible shortcomings and problems are done with collective effort, and individual challenges are less likely to happen.

Regarding the positive role of mutual respect among staff, as an effective factor in reducing job stress and facilitating collaboration, one of the emergency department participants stated, “There is more collaboration between those who respect each other.“

Concerning the familiarity and the professional and friendly behaviors of the staff in these two departments, as another important factor facilitating collaboration, one nurse from the emergency department claimed, “When the staff are friendly and sympathetic, there is no conflict. This is not a problem for the patient. Our friendship allows us to deliver and admit patients in a shorter time since we do not pay attention to trivial matters. If there are some unaccomplished tasks, the emergency department staff will take care of them so that everything works better.” Another participant said, “There is much more collaboration between friends than between those who do not know each other.“

In the busy and stressful conditions of the emergency room and hospital, professional, respectful, and friendly behaviors along with reduced tension and conflict increase collaboration and mutual understanding.

### 3. Infrastructures and processes

Infrastructures and processes were the third category and the most important factor influencing collaboration between pre-hospital and emergency departments. The availability or unavailability of specific infrastructures and work processes was one of the facilitators or barriers to inter-sectorial collaboration. This category was sub-classified as “adequate and similar equipment” “and “deficiencies in work processes.“

A. Adequate and similar equipment.

The availability of appropriate communication facilities was important since entering accidents scenes to the end of the patient transportation process. The lack of equipment and communication standards between the pre-hospital departments and emergency departments was a noticeable barrier to collaboration. The lack of wireless communication between the ambulance and the hospital as well as the lack of a direct telephone line from the pre-hospital emergency headquarters to the hospital was another example of such inadequacies.

One EMT from the pre-hospital emergency department said, “I do not have the opportunity to directly connect to the hospital from the accident scene. I cannot report the number of injured and the type of injuries to the hospital from the accident scene for them to be prepared. Thus, the onset of their treatment is delayed”. According to the participants, the lack of communication equipment for coordination between the pre-hospital departments and emergency department was a problem that seriously disrupted the patient’s rapid transport to the hospital and preparedness for collaboration.

Regarding the importance of the equipment, since pre-hospital and hospital emergency departments are managed separately in Iran, and given that pre-hospital emergency staff sometimes have to supply and replace the equipment consumed for the injured in the hospital emergency room, the existence of sufficient facilities and equipment needed by the injured and the availability of similar equipment in both sectors are important factors facilitating collaboration among staff. In this regard, one emergency medical technician asserted, “Having enough equipment in the emergency room of the hospital removes the collaboration problems; whenever I take a patient, there are enough empty beds and backboards, so I hand over the injured easily and the emergency room staff prepare a backboard for me. In this way, there is no conflict”.

The adequacy and uniformity of equipment and consumables, sufficient number of emergency experts, and recovery beds in the emergency room were important factors facilitating the handover of road accident victims.

B. Defects and disorganizations.

Patient handover and evolution are among the most important pillars of interpersonal collaboration. However, the inadequacy of processes such as documentation, development of clinical guidelines, handover of the injured from traffic incidents, delivery of consumables as well as time constraints played the role of a barrier for optimal collaboration.

One of the participants from the pre-hospital emergency department stated, “The form containing the written emergency report and patient’s history does not contain a number of important issues. For example, no blood sugar level is included for a patient with a low level of consciousness. Besides, all patients’ information is not recorded”.

The defects in the current pre-hospital emergency forms were due to the absence of some important records and patient information, and inconsistent reporting during patient handovers caused disruptions in collaboration.

Another case was the lack of coding regarding work processes and clinical guidelines. One of the nurses working in the emergency department noted, “In general, the hospital does not have a specific protocol for the delivery of emergency patients taken by ambulance, even the beds are not classified according to the triage level to locate, for example, red and green patients for further diagnoses”.

The absence of a defined protocol or a specific person in charge of admitting injuries imposes extra waiting on technicians, prolongs the delivery time, interferes with personnel duties in these two sectors, and negatively affects collaboration. Another problem in the rapid handover of injured patients was replacing pre-hospital emergency equipment consumed for the patient at the accident scene and during the delivery by emergency department equipment.

One of the emergency medical technicians from the pre-hospital emergency sector mentioned, “There is no one in charge of receiving the supplies and delivering the consumed equipment to us. I have to wait for a letter to the pharmacy. At the pharmacy, I can receive the commodity after hearing complaints and questions like: Who wrote the letter? Why do you want it? For whom was it used? It is a waste of time; I cannot answer the questions posed by the pharmacy staff since my focus was on saving the patient!“ Another participant asserted, “Another barrier to collaboration is the delivery of equipment. I myself have to go to the pharmacy and waste a lot of time to receive them, since no one is in charge of doing the same task or the equipment could be available in the triage ward so that we do not waste our time.“

Incomplete delivery of consumables, as well as wasting technician’s time in the hospital, and the creation of an environment full of tension is another barrier to collaboration between the pre-hospital department and emergency department.

A time limit is set for patient delivery by the pre-hospital emergency staff. This means that the personnel have to deliver injured patients from the pre-hospital emergency department to the emergency department staff within 10 min. The requirement to comply with this time limit has posed a lot of stress on the pre-hospital emergency staff and interrupted their collaboration with the hospital staff. An emergency medical technician states, “They have set a maximum of 10 minutes for me to stay at the hospital. When it takes longer, I will receive an alert. If the patient is in critical condition with multiple traumas, I need double-checking because I have to explain many points to the doctor and nurses. Sometimes, I have a heart attack patient, so I have to stay longer and help him to be relocated and handed over; however, I cannot collaborate appropriately with the triage department since my 10 minutes is over and I am stressed out to get back to the station early.“

## Discussion

Data analysis revealed the facilitators and barriers to the collaboration between pre-hospital and emergency departments in the case of traffic incidents in Iran. They were classified into three categories: individual capabilities, development of mutual understanding, and infrastructures and processes. These categories represent the significant role of individual and organizational factors in creating the facilitators and barriers to collaboration.

The participants believed that the staff who were more successful in acquiring knowledge and experience, had better collaboration. Higher education and more hands-on experience created more effective collaboration between pre-hospital and emergency departments. These findings were consistent with Bost’s et al.’s findings indicating that the knowledge, experience, and capability of personnel are important factors affecting collaboration [22]. Oen et al. also pointed out the personnel’s lack of knowledge as the cause of patient handover problems and as one of the challenges facing collaboration [14]. One of the challenges to collaboration was the lack of experience, as Ace and Apkar noted [23, 24].

The findings indicated that the development of mutual understanding along with a joint educational program, sharing experiences, and the occurrence of empathic behaviors were among the facilitators of collaboration as emphasized by the participants. In line with these findings, Jensen et al. also reported that the development of mutual understanding improves the quality of collaboration in patient delivery [25]. The results of the study conducted by Bruce et al. also highlighted the positive role of joint educational programs in reducing the risks of patient handover [26]. The participants stated that the existence of shared knowledge and experiences helps to better understand shared experiences with patients, the workplace, and medical interventions at the accident scene and hospital, leading to improved collaboration.

One example of mutual understanding is empathic behaviors. This factor contributed significantly to increase collaboration. Professional, friendly, supportive behaviors and previous knowledge about each other would increase encouragement, trust and mutual relationship among colleagues. In this case, the collaboration is facilitated and problems are solved with a better and faster collective effort. The findings of this study are confirmed by those obtained by Beh Nia et al., who believed that poor intragroup communication and mistrust are challenges to collaboration [27]. Dawson et al. also described staff encouragement as a positive factor in improving patient handover between pre-hospital emergency and emergency department medical staff [9]. Other researchers have also emphasized on the critical role of trust in facilitating and enhancing collaboration and reducing mistakes [28, 29].

The results indicated that if infrastructures were provided and the processes were defined and formulated, they will facilitate collaboration; otherwise, they prevent collaboration. Establishing a timely, accurate, and professional communication is the basis of establishing a mutual collaboration. The results of this study showed that pre-hospital emergency personnel suffered from lack of communication facilities and believe that this deficiency affects patient care, reporting, and coordination. In a qualitative study, Khademian et al. investigated the effective factors in improving teamwork in a trauma center. They also announced the inefficiency of the information sharing system as a barrier to optimal teamwork [30]. Studies by Miyers et al. highlighted the relationship and coordination between pre-hospital emergency information systems and other relevant information systems to provide appropriate pre-hospital emergency services [31]. The role of communication equipment as one of the most influential components in the performance of Iran’s pre-hospital emergency department is similar to the results found by Adent. In his study, the preferred key feature of the French pre-hospital emergency department was introduced to be the well-suited communication equipment system [32]. On the other hand, technology has been served the health systems in different countries. A review of pre-hospital emergency systems in developed countries shows that online medical communication is currently one of the popular features used and plays a fundamental role in providing services to far-reaching areas, that is, the principle of equality in access to services and communication acceleration. In Iran, however, the technological progress in the pre-hospital emergency departments is not similar to that observed in the developed countries and does not have such features [32–36].

In the case of other infrastructures, the availability of equipment and the similarity of equipment and supplies in the pre-hospital and emergency departments was one of the most influential factors for inter-sectorial collaboration. Mock et al. found the significant effect of physical resources on the care provided for traffic accidents [12]. In Vitkaitis’ study, the most important problems were emergency services in Lithuania, old ambulances, and the lack of integrated standards for medical education, which was emphasized as one of the factors affecting the poor performance of emergency medical services in this country [37]. These results are in line with the findings of the current study, indicating that pre-hospital and emergency department personnel are considered as specialists and capital in each country and their time is valueless. If they have access to sufficient infrastructure and facilities, they will have better collaboration, services, and will be more successful in satisfying patients.

According to the findings, the infrastructures and processes play an important role in effective collaboration; plus, their inadequacy causes problems in proper collaboration. Deficiencies such as lack of programming, specific processes for patient handover, equipment, reporting, and documentation are considered to be important barriers to effective collaboration. Accordingly, there is no specific protocol for admitting patients to the hospital and this had a negative impact on inter-sectorial collaboration. Bahadori et al. studied collaboration in crises and called detailed processes to achieve maximum collaboration [38]. As Bost et al. noted, clinical handover of patients is an important process that can help or prevent the safe transfer of patients to health systems [22]. Poor intragroup communication, mistrust, and lack of teamwork processes are among challenges facing collaboration [27]. Mizell et al. also assumed the standardization of handover procedures and training interdisciplinary issues as one of the important factors in enhancing collaboration between pre-hospital and emergency departments [39]. In Vitkaitis’ study, the lack of unified standards was considered as one of the negative factors regarding the performance of emergency medical services in Lithuania [37]. This finding was in line with the results of this study. Complete documentation and reporting are the major factors in the handover of traffic accident victims between the pre-hospital and emergency departments. The lack or shortage of necessary equipment and infrastructures for collecting and presenting patient information has posed challenges to inter-sectorial collaboration.

## Limitations

The present study was a qualitative study with the aim of identifying the facilitators and barriers of collaboration between pre-hospital and emergency departments to handover the injured from traffic accidents in Iran. One of the limitations of this study was the qualitative feature of it; therefore, the results cannot be generalized to other emergency centers. Another limitation was the small sample size we used in this study. Moreover, the data collection was limited to a specific region, which cannot be indicative of other emergency centers in the whole country.

More quantitative and qualitative studies with a larger sample size are suggested in other emergency centers in other provinces of the country in order to better identify the facilitators and barriers to collaboration using the experiences of other staff across the country.

## Conclusion

The current study identified the main facilitators and barriers to the traffic accidents injured delivery from pre-hospital emergency sectors to the hospitals in Iran. The results of this study indicate that individual capabilities such as knowledge and experience, development of mutual understanding through joint educational programs, sharing experiences, empathic behaviors, efficient infrastructures and processes such as the existence of specific work processes, documentation and paying attention to its prerequisites, and the availability of adequate and similar equipment are essential components in improving and facilitating collaboration between the pre-hospital and emergency departments. Ignoring the mentioned factors are barriers resulting in increased mortality rates and injuries caused by road accidents. Considering the undeniable effects of collaboration on the quality of medical services provided to traffic accident victims and the important challenges of this stage, issues related to the patient handover should be concerned as a part of the pre-hospital and emergency staff educational programs. Health managers and policy makers should take measures to develop policies and programs to strengthen facilitators and solve the challenges of establishing an appropriate collaboration framework. Empowering facilitators and removing barriers can provide positive and effective grounds for establishing collaboration in order to improve health services for road accident victims.

## Data Availability

The datasets used and/or analyzed during the current study are available from the corresponding author upon reasonable request.
